# Fibroblast growth factor receptors as targets for anticancer therapy in cholangiocarcinomas and urothelial carcinomas

**DOI:** 10.1016/j.heliyon.2023.e19541

**Published:** 2023-08-27

**Authors:** Demi Wekking, Andrea Pretta, Serafina Martella, Alessandra Pia D'Agata, Joanna Joeun Choe, Nerina Denaro, Cinzia Solinas, Mario Scartozzi

**Affiliations:** a Amsterdam UMC, Location Academic Medical Centre, University of Amsterdam, Amsterdam, the Netherlands; b Medical Oncology Unit, University Hospital and University of Cagliari, Italy; c Medical Oncology, University Hospital Policlinico G.Rodolico-San Marco, 95123, Catania, Italy; d Cancer Outcomes Research and Education, Massachusetts General Hospital, Boston, MA, USA; e Medical Oncology, Policlinico Milano, Italy; f Medical Oncology AOU Cagliari Policlinico Duilio Casula, Monserrato, CA, Italy

**Keywords:** Fibroblast growth factor receptor inhibitors, Cholangiocarcinoma, Bladder cancer, FGFR genetic alterations, Targeted therapy, Acquired resistance

## Abstract

Cholangiocarcinomas and urothelial carcinomas are lethal tumors worldwide and only a minority of patients are eligible for surgery at diagnosis. Moreover, patients are poorly responsive to current therapeutic strategies, including chemotherapy, radiotherapy, immunotherapy, and multimodality treatments. Recently, several advances have been made in precision medicine and these results are modifying the treatment paradigm for patients diagnosed with cholangiocarcinomas and urothelial carcinoma. These histotypes exhibit a high rate of multiple fibroblast growth factor receptor (FGFR) genetic alterations and numerous preclinical and clinical studies support FGFR as a highly attractive novel therapeutic target. Moreover, identifying specific genetic alterations may predict the tumor's response to conventional and novel FGFR-targeted drugs. Recent clinical studies showed promising data for FGFR-targeted therapy in reducing tumor volume and led to the United States Food and Drug Administration (FDA) approval of, e.g., pemigatinib, infigratinib, futibatinib, and erdafitinib. Moreover, FGFR inhibitors show promising results in the first-line setting of cholangiocarcinomas and urothelial carcinomas. Pemigatinib (FIGHT-302) and futibatinib (FOENIX-CAA3) are being evaluated in phase III trials that compare these agents to current first-line gemcitabine and cisplatin in *FGFR2*-rearranged cholangiocarcinoma. However, complexity in targeting the FGFR signaling pathway is observed. Herein, we describe the characteristics of the FDA-approved and other investigational FGFR-targeted therapeutics, evaluate the most recent preclinical and clinical studies focusing on targeting *FGFR* genomic alterations in the treatment of cholangiocarcinomas and urothelial cancer, and provide insight into factors involved in response and (acquired) resistance to FGFR inhibition.

## Highlights

• FGFRs are targets for novel oral tyrosine kinase inhibitors in cholangiocarcinoma and urothelial carcinomas.
• Superior responses to FGFR inhibitors are observed in cholangiocarcinomas exhibiting *FGFR2* fusions and rearrangements.
• The best activity of FGFR inhibitors in urothelial cancers is demonstrated in tumors harboring *FGFR2* or 3 mutations.
• Pemigatinib, infigratinib, and futibatinib are approved for cholangiocarcinoma positive for *FGFR2* fusions or rearrangements.
• Erdafitinib is approved for first-line treatment in urothelial cancers exhibiting *FGFR2* or 3 mutations.

## 1 Introduction

Cholangiocarcinoma and urothelial carcinomas are major global health concerns, demonstrating a 5-year survival rate of 2% and 6% in advanced and metastatic stages, respectively [1,2]. The incidence is still slightly rising and the prognosis remains poor [3]. Currently, complete cystectomy with negative margins and complete resection of the cholangiocarcinoma remains the only potentially curative treatment; however, only a minority of patients are eligible for surgery due to the presence of advanced-stage disease at the time of diagnosis [4,5]. The standard of care for patients diagnosed with locally-advanced or metastatic cholangiocarcinoma consists of systemic chemotherapy with gemcitabine and cisplatin and with durvalumab. Patients with metastatic urothelial cancer receive platinum-based chemotherapy [4].

Fibroblast growth factors (FGFs) and their receptors (FGFRs), a family of transmembrane receptor tyrosine kinases (FGFR1-4), are involved in an abundance of intracellular events that promote survival and proliferative signaling pathways [6,7]. FGF-FGFR signaling is triggered by ligand-dependent receptor dimerization upon binding of FGF to the cell surface. This leads to intracellular phosphorylation of receptor kinase domains, an intracellular signaling cascade, and gene transcription that activates many intracellular survival and proliferation pathways. Aberrant activation of FGFR pathway signaling is observed in subgroups of patients diagnosed with distinct malignancies. Amplifications, fusions, and missense mutations in *FGFRs1-4* exons, as well as changes in noncoding regions, aberrant epigenetic or transcriptional regulation, and alterations in dynamic interplay in tumor microenvironment mediate enhanced or constitutively-activated FGFR pathway signaling. This deregulation influences critical pathways for cell proliferation, survival and drug resistance, angiogenesis, metastasis, and immune evasion [3,7,8]. Some of these are the Ras-Raf-MEK-ERK pathway, the PI3-AKT-mTOR pathway, and the JAK-STAT pathway [9]. Therefore, targeting this pathway represents an attractive therapeutic strategy.

Biliary tract cancers include a heterogeneous group of malignancies that can be divided into intrahepatic cholangiocarcinoma (iCCA), perihilar cholangiocarcinoma (pCCA), distal cholangiocarcinoma (dCCA), and gallbladder cancer [3,5]. The clinical spectrum of urothelial cancer comprises non-muscle invasive bladder cancer (NMIBC), muscle-invasive bladder cancer (MIBC), and upper urinary tract carcinoma [4]. Biliary tract and bladder carcinomas have emerged as candidates for personalized medicine with the advent of genomic profiling. For instance, next generation sequencing (NGS) of DNA/RNA and immunohistochemistry are able to identify multiple clinically-relevant genetic alterations, including *FGFR* rearrangements, fusions, and mutations.

*FGFR* mutations occur in approximately 7% of all cancers and are particularly common in urothelial and cholangiocarcinoma [8]. Approximately 50% of bladder cancers harbor *FGFR* genetic alterations; 75% of the NMIBC and approximately 15% of high-grade invasive urothelial cancers harbor *FGFR3* genetic alterations, and 2% of urothelial cancers carry *FGFR2* mutations [6]. Unlike urothelial cancers, cholangiocarcinoma demonstrates high rates of *FGFR2* fusions or rearrangements. *FGFR2* fusions have been found in up to 11–45% of patients diagnosed with iCCA, possibly associated with a favorable prognosis [6,7,10]. Recent preclinical studies with FGFR inhibitors support the potential of these genetic alterations as a therapeutic target [[11], [12], [13], [14]]. Several FGFR inhibitors demonstrated activity in cholangiocarcinoma and urothelial cancer harboring *FGFR2* and *FGFR3* alterations.

Current front-line therapy consisting of chemotherapy with gemcitabine and cisplatin and with durvalumab offers a median progression-free survival (mPFS) of 7.2 months and overall survival (OS) of 12.8 months in all comers with cholangiocarcinoma [15]. Interestingly, a mPFS of 9 months was observed for futibatinib as second-line or later-line treatment, a mPFS of 7.3 months for infigratinib as second-line or later-line treatment, and a mPFS of 7.0 months for pemigatinib as second-line or later-line treatment in indolent FGFR-exhibiting tumors. In urothelial carcinoma, an OS of 14–15 months is achieved in after first-line cisplatin-based chemotherapy. With erdafitinib as second-line treatment, a mPFS of 5.5 months and mOS of 13.8 months was observed. Pemigatinib is the first United Stated Food and Drug Administration (FDA)-approved FGFR inhibitor for *FGFR2* fusion in cholangiocarcinoma [10]. Recently, infigratinib and futibatinib received approval from the FDA for this cancer as well. Erdafitinib was approved as a first-line treatment of urothelial tumors bearing *FGFR2/3* fusion or mutants and for second-line therapy of metastatic or unresectable urothelial carcinoma. These encouraging outcomes are promising in implementing FGFR inhibitors in first-line treatment for FGFR-harboring tumors.

However, FGFR-targeted treatments are only approved for a subset of tumors with specific genetic alterations. Moreover, in these subsets, primary resistance is observed and acquired resistance emerges following treatment. This highlights the complexity of targeting the FGFR signaling pathway and therefore clinical variability in treatment response.

In this review, we describe the characteristics of the FDA-approved and other investigational FGFR-targeted therapeutics, evaluate the most recent pre and clinical studies focusing on targeting *FGFR* genomic alterations in the treatment of cholangiocarcinoma and urothelial carcinoma, and provide insight into factors involved in response and (acquired) resistance to FGFR inhibition.

## 2 FGFR as an actionable target for precision medicine

Molecular testing revealed a broad spectrum of *FGFR* gene alterations that resulted in the development of various FGFR-targeted therapies. These alterations may respond differently to FGFR inhibitors and therefore demonstrate different outcomes. Selection of the type of fusions is thus critical to predicting the responsiveness to treatment. For instance, fusions retaining the tyrosine kinase domain are sensitive to FGFR inhibitors. Moreover, some inhibitors, such as infigratinib (BGJ398) and AZD4547, showed strong activity in sub-classical and classical *FGFR2* fusions due to the complete blockade of activation by phosphorylation of all downstream signals [16]. The selectivity of the inhibitor drug predicts sensitivity as well. For instance, pan-FGFR inhibitors inhibit the autophosphorylation in tumor cells, thereby having anti-proliferative properties and thus reducing cell proliferation and growth.

Small molecule inhibitors of FGFR receptor kinases are classified into FGFR1-3 inhibitors, FGFR4 inhibitors, pan-FGFR inhibitors, or multi-kinase FGFR inhibitors (non-selective). First-generation FGFR inhibitors are non-selective tyrosine kinase inhibitors (ponatinib, nintedanib, or dovitinib) that target the ATP-binding cleft of the protein kinase domains of several growth factor receptors such as VEGFR and PDGFRs (Table 1). This non-selectivity prevents the establishment of resistance mechanisms [6,17].

**Table 1:** Overview of the discussed FGFR inhibitors.

FGFR inhibitor	Type of inhibitor	Type of tumor in trials
Pemigatinib (INCB054828)	Selective FGFR1-3 inhibitor	Cholangiocarcinoma and urothelial carcinoma
Futibatinib (TAS-120)	Type VI pan-FGFR inhibitor	Cholangiocarcinoma
Infigratinib (BJG398)	Type I1/2A FGFR1-3 inhibitor	Cholangiocarcinoma and urothelial carcinoma
Erdafitinib (JNJ-42756493)	Type I1/2A pan-FGFR inhibitor	Cholangiocarcinoma and urothelial carcinoma
Derazantinib (ARQ 087)	ATP-competitive multi-kinase pan-FGFR inhibitor	Cholangiocarcinoma and urothelial carcinoma
Dovitinib (TKI258)	Type I1/2B multi-kinase FGFR1-3 inhibitor	Urothelial carcinoma
Rogaratinib (BAY1163877)	Pan-FGFR1-4 inhibitor	Urothelial carcinoma
Zolagritinib (Debio 1347)	Type I1/2B FGFR1-3 inhibitor	Cholangiocarcinoma
Ponatinib (AP24534)	Ttype IIA multi-kinase	Cholangiocarcinoma
RLY-4008	Selective potent next-generation FGFR2	Cholangiocarcinoma
AZD4547	Type I1/2A FGFR1-3 inhibitor	Urothelial carcinoma
LY2874455	TypeI1/2B pan-FGFR inhibitor	Urothelial carcinoma
Nintedanib (BIBF1120)	Type I multi-kinase inhibitor	Urothelial carcinoma

**Fig. 1:**
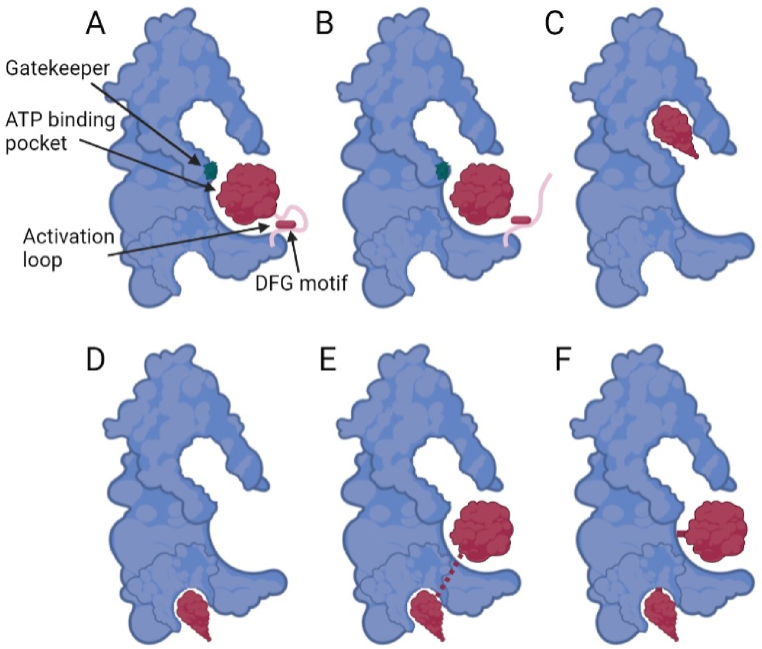
FGFR kinase inhibitors. Type I inhibitor in DFG_in_ active form (A), Type II in DFG_out_ inactive form (B), Type III allosteric binding within the ATP binding pocket (C), Type IV allosteric binding (D), Type V bivalent binding (E), and Type VI covalent binding (F). Figure created on BioRender.

FGFR inhibitors can be further classified into ATP-competitive (type I, I1/2A, I1/2B, II), allosteric (III, IV), bivalent (V), and covalent inhibitors (VI) [18]. Type I inhibitors target the kinase adenine-binding site in its active DFG-D_in_ structure (Fig. 1A). Type I1/2 antagonists target an inactive enzyme with a DFG-D_in_conformation. Type II inhibitors target the kinase in its DFG-D_out_ conformation (Fig. 1B). Type III inhibitors target an allosteric site between the N-terminal and C-terminal lobes in a conformation that does not preclude ATP binding (Fig. 1C). Type IV antagonists bind outside the regions between the two lobes (Fig. 1D). Type V inhibitors span two portions of the kinase domain (Fig. 1E). Type VI forms a covalent bond with its target enzyme (Fig. 1F). Irreversible inhibitors (i.e. futibatinib (TAS-120)) bind more strongly with their target protein. These covalent inhibitors confer enhanced selectivity and binding kinetics compared to non-covalent antagonists [19]. Subtype A inhibitors extend past the gatekeeper into the hydrophobic cleft, while subtype B inhibitors do not extend into the back cleft [18]. Inhibitors that extend past the gatekeeper result in a longer residence time.

The next generations of FGFR tyrosine kinase inhibitors selectively target the ATP-binding cleft of the kinase domain of the FGFR [6,10].

## 3 Clinical outcomes for patients with urothelial and intrahepatic cholangiocarcinoma receiving FGFR inhibitors

Multiple FGFR inhibitors are under investigation in clinical trials and support their potential as first- or later-line treatment. Four inhibitors are already FDA-approved for treating these tumors and their process of approval will be discussed as well as outcomes from ongoing studies. Additionally, outcomes from studies evaluating other FGFR inhibitors will be reviewed as well.

### 3.1 Pemigatinib (INCB054828)

Pemigatinib, a selective FGFR1-3 inhibitor, is FDA-approved for patients previously treated with systemic therapy diagnosed with unresectable, locally advanced, or metastatic cholangiocarcinoma who have *FGFR2* fusion or other rearrangements [20].

A phase II clinical trial FIGHT-202 (NCT02924376) demonstrated an objective response rate (ORR) of 37.0% (95% CI, 27.9–46.9) of pemigatinib as second or later-line treatment in patients (n = 108) diagnosed with advanced/metastatic cholangiocarcinoma and *FGFR2* fusions or rearrangements [21]. A median duration of response (mDOR) of 8.1 months (95% CI, 5.7–13.1) was achieved. The mPFS was 7 months (95% CI, 6.1–10.5), the median overall survival (mOS) was 17.5 months (95% CI, 14.4–22.9), and the disease control rate (DCR) was 82.4% (95% CI, 73.9–89.1). Despite these impressive responses of pemigatinib in these groups of patients, results in patients harboring *FGFR* amplification or mutations have been disappointing [7,22,23]. Another phase II study (NCT04256980) was conducted to evaluate pemigatinib in patients (n = 30) with advanced or metastatic cholangiocarcinoma bearing *FGFR2* fusions or rearrangements who progressed on systemic therapy. The study demonstrated an ORR of 50% (95% CI, 31.3–68.7), a DCR of 100% (95% CI, 88.4–100), and a mPFS of 6.3 months (95% CI, 4.9-not estimable) [24]. Both studies had absence of a comparator arm, which makes it difficult to assess the relative effectiveness of pemigatinib. The phase III study FIGHT-302 (NCT03656536) is comparing the efficacy and safety of pemigatinib to gemcitabine plus cisplatin chemotherapy in unresectable or metastatic cholangiocarcinoma. Another study, a phase I trial (NCT04088188), is evaluating the efficacy of pemigatinib in combination with gemcitabine and cisplatin.

Pemigatinib is studied in trials enrolling patients with urothelial carcinoma as well. A phase II study FIGHT-201 (NCT02872714) was conducted to evaluate pemigatinib in patients (n = 64) with metastatic or surgically-unresectable urothelial carcinoma with *FGFR3* mutations or fusions progressed after systemic treatment. An ORR of 25% (95% CI, 14–40) was achieved, with an mPFS of 4.1 months [25]. However, the long-term efficacy and potential survival benefits of pemigatinib in these patients are not yet determined. Another phase II study PEGASUS (NCT04294277) evaluates pemigatinib in patients with high-risk urothelial cancer who underwent radical surgery. Lastly, a phase II study (NCT03914794) is currently recruiting patients with urothelial carcinoma to evaluate the efficacy of pemigatinib.

### 3.2 Futibatinib (TAS-120)

Futibatinib, a type VI pan-FGFR inhibitor, is recently FDA-approved for previously-treated, unresectable, locally-advanced, or metastatic iCCA with *FGFR2* fusions or other rearrangements [26]. The antagonist is highly selective and covalently binds to FGFR2 and interacts with the signaling pathway by inhibiting FGFR phosphorylation.

A phase I FOENIX-101 (NCT02052778) study demonstrated an ORR of 16.7% (95% CI, 7.0–31.4), mDOR of 6.9 months, and a DCR of 78.6% (95% CI, 63.2–89.7) in patients (n = 42) with iCCA exhibiting *FGFR2* fusions or rearrangements. A mPFS of 6.0 months (95% CI, 3.7–9.0) was achieved [27]. In this study, some fusions with novel partner genes might not have been detected in tumors harboring *FGFR2* rearrangements assays. Comutations may influence the sensitivity to futibatinib, thereby affecting the trial outcomes. Furthermore, the phase II study FOENIX-CCA2 (NCT02052778) evaluated futibatinib in patients (n = 103) diagnosed with iCCA with *FGFR2* fusions (n = 80) or other rearrangements (n = 23), refractory on systemic therapy, and demonstrated an ORR of 41.7% (95% Cl, 32–52), a DCR of 82.5% (95% CI, 74–89), mDOR of 9.7 months (95% Cl, 7.6–17.1) and a mPFS of 9.0 months (95% CI, 6.9–13.1). A mOS of 21.7 months (95% CI, 14.5-not estimated) was achieved [28]. These data were consistent with the study's preliminary analysis and reinforced the efficacy of futibatinib in patients with iCCA exhibiting *FGFR2* fusions or other rearrangements [29]. The treatment effect of futibatinib was not accurately determined due to a lack of a comparator arm. The phase III trial FOENIX-CCA3 (NCT04093362) aims to provide insights into this treatment efficacy and its role in the first line setting.

A treatment comparison was performed using patient data from FIGHT-202 (n = 107, pemigatinib) and FOENIX-CCA2 (n = 103, futibatinib) and demonstrated a significantly lower risk of progression or death when patients were treated with futibatinib compared to chemotherapy [30]. No statistically-significant differences were found in treatment outcomes between futibatinib and pemigatinib; however, numerical benefits for futibatinib were observed. These outcomes suggest a more prolonged survival of futibatinib compared to chemotherapy among advanced iCCA-bearing *FGFR2* fusions or rearrangements before receiving systemic treatment. A study (NCT04507503) is providing expanded access to futibatinib for patients diagnosed with advanced cholangiocarcinoma exhibiting *FGFR2* rearrangements.

### 3.3 Infigratinib (BJG398)

Infigratinib, a type I1/2A FGFR1-3 tyrosine kinase inhibitor, received FDA approval for previously-treated, unresectable, locally-advanced or metastatic cholangiocarcinoma exhibiting *FGFR2* fusions or other rearrangements [31]. Of interest, the European Medicines Agency withdrew the application for further development of infigratinib since a phase II study (NCT02150967) did not demonstrate that the benefits of the agent outweigh its risks. Therefore, at the time of writing, the sponsor intends to halt the ongoing phase III PROOF-301 (NCT03773302), in which infigratinib is evaluated as a potential first-line treatment in advanced or inoperable cholangiocarcinoma harboring *FGFR2* gene fusions or translocations [32]. Moreover, the phase III trial (NCT04197986) PROOF-302 is terminated due to the discontinuation of the development of infigratinib as well.

In one study, tumor volume reduction was observed in a patient with cholangiocarcinoma harboring *FGFR2* fusions in response to BGJ398 [33]. Subsequently, the agents were evaluated as a second or later-line treatment in a phase II (NCT02150967) study that included patients (n = 71) diagnosed with advanced iCCA harboring *FGFR2* fusions. The study demonstrated an ORR of 26.9% (95% CI, 16.8–39.1), mPFS of 6.9 months (95% CI, 5.3–7.6), and an OS of 12.5 months (95% CI, 9.9–16.6) [34]. The update of the study demonstrated an ORR of 25.0% (95% CI, 23.1–32.2) in patients (n = 108) with advanced/metastatic cholangiocarcinoma with *FGFR2* alterations (81% in-frame *FGFR2* fusions, 19% had other *FGFR2* rearrangements). The mDOR was 5.0 months (95% CI, 3.7–9.3), the mPFS was 7.3 (95% CI, 5.6–7.6), and the DCR was 91.0% (95% CI, 84.3–90.6). A mOR of 12.2 months (95% CI,10.7–14.9) was reached [35]. The tumor responses to infigratinib were blinded and independently assessed to minimize the risk of bias that might arise in an open-label design.

Despite that these results suggest that infigratinib has activity in cholangiocarcinoma harboring *FGFR2* fusions previously treated with systemic therapy, the discontinuation of the development of infigratinib precludes the implementation of the agent in first-line treatment.

Infigratinib is evaluated in urothelial carcinoma as well. A phase I study (NCT01004224) evaluated infigratinib in patients (n = 67) with urothelial cancer harboring *FGFR3* alterations. The study demonstrated an ORR of 25.4%, mPFS of 3.75 months (95% CI, 3.09–5.39), and an OS of 7.75 months (95% CI, 5.65–11.60) [36]. When anatomically divided, disease control was observed in 59.3% of the patients (n = 59) with *FGFR3* mutations and/or fusions in the urinary bladder. In this group, an ORR of 22%, a mPFS of 3.65 months (95% CI 2.76–4.91 months), and a mOS of 7.0 months (95% CI 5.52–11.53) were observed [37]. In patients (n = 8) with *FGFR3* mutations and/or fusions in the upper tract, an ORR of 50% and a DCR of 100% were observed. Moreover, the study demonstrated a mPFS of 8.54 months (95% CI, 3.68–12.91) and a mOS of 21.82 months (95% CI 11.63-not estimable). These outcomes suggest more significant activity of infigratinib in the upper tract. However, the study's findings should be interpreted with caution, as the observed outcomes could be affected by confounding variables. The authors were limited in their ability, due to a small sample size, to account and correct for these clinical and demographic variables that could influence the data.

The greater activity of infigratinib in patient with localized upper tract urothelial carcinoma is supported by an interim analysis of a phase Ib study (NCT04228042). Herein, an ORR of 44% (n = 9) was observed when giving infigratinib prior to surgery [38]. Nevertheless, caution is warranted when interpreting the data due to the amount of patients included.

### 3.4 Erdafitinib (JNJ-42756493)

Erdafitinib, type I1/2A pan-FGFR inhibitor, is FDA-approved for treating locally-advanced, unresectable, or metastatic urothelial carcinoma that has progressed during or after platinum-based chemotherapy and as first-line treatment of urothelial bladder cancer exhibiting *FGFR2* or 3 mutations [39].

Patients with urothelial tumors with *FGFR2* truncation or fusion showed treatment responses in a phase I study evaluating JNJ-42756493 [40]. Subsequently, erdafitinib was evaluated in a phase I trial (NCT01703481). ORR of 46.2% and 27.3% were achieved in patients with urothelial carcinoma (n = 26) and cholangiocarcinoma (n = 11) with *FGFR* alterations, respectively [41]. A mDOR of 5.6 and 11.4 months was achieved for urothelial carcinoma and cholangiocarcinoma, respectively. The mPFS was 5.1 months for patients with urothelial carcinoma. However, no conclusions were made on which *FGFR* variants correlated with response to the agent because of the small sample sizes.

Another global phase II study BLC2011 (NCT02365597) included patients (n = 99) with urothelial cancer harboring *FGFR* alterations, previously treated with chemotherapy or ineligible for cisplatin, and chemotherapy *naïve*. Among them, 74 were carrying *FGFR3* mutations and 25 had *FGFR2/3* fusions. The confirmed ORR was 40% (95% CI, 31%–50%), 3% complete, and 37% partial responses. Among patients exhibiting *FGFR3* mutations (n = 74), a 49% response rate was achieved for erdafitinib compared to 16% in patients with *FGFR* fusions (n = 25). For patients (n = 22) treated with immune checkpoint inhibitors (ICIs) prior to treatment with erdafitinib, the confirmed ORR was 59%, mPFS was 5.5 months (95% CI, 4.2–6 months), and mOS was 13.8 months (95% CI, 9.8-not reached) [42,43]. These findings led to FDA approval of erdafitinib for previously-treated *FGFR3*-altered urothelial carcinoma. Median efficacy follow-up of 24.0 months of BLC2011 study (NCT02365597) in patients (n = 101) with locally-advanced or metastatic urothelial carcinoma with second-line erdafitinib was evaluated (69% had *FGFR* mutations, 25% harbored fusions, 6% had both) [44]. The ORR was 40% (95% CI, 30–49), of which 4% reached a complete response. The overall DCR was 80% (95% CI, 72–88), the mDOR was 6.0 months (95% CI, 4.2–7.5), and mPFS was 5.5 months (95% CI, 4.3–6.0). The 12-month PFS rate was 21% (95% CI,13–29), the mOS was 11.3 months (95% CI, 9.7–15.2), and the 24-month OS rate was 31% (95% CI,22–40) [44]. One limitation of the study is that the tumor responses to infigratinib were assessed in an open-label, single-arm, study design, making it difficult to reduce potential bias.

For patients with advanced cholangiocarcinoma, a phase IIa study demonstrated a partial response of 46.7% and stable disease of 33.3% in patients (n = 17) receiving prior treatment for cholangiocarcinoma exhibiting FGFR alterations (10 FGFR2 fusions, 4 *FGFR3* mutations, 1 *FGFR3* fusion, and two *FGFR3* mutations). The ORR was 47% and DCR was 80% (n = 15) [45]. Moreover, a phase IIa study LUC2001 (NCT02699606) demonstrated an ORR of 50% in patients with advanced cholangiocarcinoma exhibiting *FGFR* alterations (*FGFR2* fusions n = 8, *FGFR2* mutations n = 3, *FGFR3* fusion n = 1, *FGFR3* mutations n = 2) treated with erdafitinib, who progressed on systemic treatment [46]. Solely analyzing *FGFR2* fusions, an ORR of 60% and a DCR of 100% were achieved. The overall study population achieved a mPFS of 5.59 (95% CI, 1.87–13.67), mDOR of 6.83 (95% CI, 3.65–12.16), and DCR of 83.3%. A mPFS of 12.35 (95% CI, 3.15–19.38) was achieved specifically for *FGFR2* alterations. Updated analysis of the LUC2001 study (NCT02699606) demonstrated an ORR of 40.9% (95% CI, 20.7–63.6), mDOR was 7.3 months (95% CI, 3.7–17.5), mPFS 5.6 months (95% CI, 3.6–12.7), and mOS was 40.2 months (95% CI, 9.9-not estimable) in patients with fusions (n = 14) and mutations (n = 8) [47]. Responses were achieved in 8 patients with *FGFR* fusions and 1 with *FGFR* mutation. However, the study's findings should be interpreted with caution because of the low sample sizes that reduces the statistical power of the findings.

Currently, the phase III (NCT03390504) trial compares erdafitinib monotherapy to chemotherapy (docetaxel or vinflunine) in patients with metastatic urothelial cancer with *FGFR* gene alterations prior to receiving anti-PD(L)1 therapy and pembrolizumab without prior anti-PD(L)1. The study outcomes can contribute to how to sequence these drugs most efficiently, based on suggestions that *FGFR* alterations are more prevalent in tumors with reduced T-cell infiltrations, indicating lower response to ICIs.

Another phase Ib-II study (NCT03473743) currently evaluates erdafitinib in patients with metastatic or locally-advanced urothelial carcinoma harboring *FGFR* genomic alterations that did not receive systemic therapy before. Moreover, a phase II study (NCT04917809) recruits patients with recurrent non-invasive bladder cancer exhibiting *FGFR3* mutations to evaluate erdafitinib. Lastly, a phase II study (NCT04172675) recruits patients with recurred NMIBC with *FGFR* mutations or fusions who received Bacillus Calmette-Guérin (BCG) to evaluate erdafitinib compared to chemotherapy.

### 3.5 Derazantinib (ARQ 087)

Derazantinib is an ATP-competitive pan-FGFR inhibitor with multi-kinase activity [48]. In a phase I study, patients (n = 10) with iCCA were treated with ARQ 087. Five patients with *FGFR2* fusions showed two confirmed PRs, a SD of 24–41 weeks was achieved, and a 25% tumor reduction was observed. Progressive disease was observed in the remaining two patients with *FGFR2* fusions and five without *FGFR* aberrations. Furthermore, a patient diagnosed with urothelial cancer with *FGFR2* amplification had a confirmed PR (35% tumor reduction) and completed 40 weeks of treatment.

A phase I/II study (NCT01752920), including patients (n = 29) with cholangiocarcinoma with *FGFR2* fusions, demonstrated an ORR of 20.7%, DCR of 82.8%, and a mPFS of 5.7 months (95% CI, 4.0–9.2) [49]. Cohort 1 of study FIDES-01 (NCT01752920) evaluated derazantinib in patients (n = 103) with iCCA with *FGFR2* fusions or rearrangements. Confirmed ORR of 21.4% (95% CI, 13.9–30.5) was reached, including 22 PRs. The DCR was 74.8% (95% CI, 66.3–83.6), the mDOR was 6.4 months (95% CI, 3.9–9.2), the mPFS was 7.8 months (95% CI, 5.5–8.2), and mOS was 15.5 months (95% CI, 11.8–21.9) [50]. Moreover, an interim analysis of derazantinib treatment in patients (n = 23) with advanced iCCA exhibiting *FGFR2* mutations or amplifications previously treated with chemotherapy demonstrated a DCR of 73.9% (95% CI, 51.6–89.8) with an mPFS of 7.3 (3.5–16.7) months [51]. The final results are awaited (NCT03230318). Despite the low sample size in the analysis, clinically meaningful anti-tumor efficacy was reported in patients with *FGFR2* mutations or amplifications.

A study (NCT04087876) provides expanded access to derazantinib for patients diagnosed with locally-advanced, inoperable, or metastatic iCCA with *FGFR* gene aberrations. Another phase I/II study FIDES-02 (NCT04045613) compares derazantinib monotherapy with derazantinib combined with atezolizumab in patients diagnosed with advanced urothelial carcinoma exhibiting *FGFR* genomic alterations.

### 3.6 Dovitinib (TKI258)

Dovitinib is a type I1/2B multi-kinase FGFR1-3 inhibitor. The drug was evaluated in a phase II study (NCT01732107) in BCG-unresponsive urothelial cancer (n = 13) harboring *FGFR3* alterations. A RR of 8% (1/10) was observed [52]. The absent anti-tumor efficacy observed in this study could potentially be attributed to the limited characterization of the tumors. The current study may have missed molecular alterations required for tumor responses. Previous trials have shown tumor responses in patients with *FGFR3* mutations or fusions. Another phase II study (NCT00790426) was conducted to evaluate TKI258 in patients with advanced urothelial cancer exhibiting *FGFR3* mutations [53]. The study was terminated since the ORR did not meet the requirements to enter the trial's next phase.

### 3.7 Rogaratinib (BAY1163877)

Rogaratinib, a pan-FGFR1-4 inhibitor, was evaluated in a phase II/III study FORT-1 (NCT03410693) comparing rogaratinib with chemotherapy in patients who progressed on prior systemic therapy with advanced metastatic urothelial carcinoma harboring *FGFR1* or *FGFR3* aberrations. Among the patients (n = 21) with *FGFR3* mutations or fusions who were treated with rogaratinib, an ORR of 52.4% (95% CI, 29.8–74.3) was achieved compared to 26.7% (95% CI, 7.8–55.1) treated with chemotherapy (n = 15) [54]. A phase I (NCT01976741) study was conducted in which rogaratinib was evaluated in patients (n = 51) with urothelial cancer expressing high FGFR3. Among them, an ORR of 24%, SD of 49%, and DCR of 73% were achieved [55]. However, only 35% of the patients had an underlying *FGFR* DNA alteration (*FGFR3* mutations n = 16, *FGFR3* fusions n = 2). In addition, the clinical validation of the cutoff values utilized to identify FGFR mRNA overexpression has not been established. Hence, the results regarding the efficacy of rogaratinib in FGFR alterations should be carefully interpreted.

### 3.8 Zoligratinib (Debio 1347)

Debio 1347 is a type I1/2B inhibitor selective for FGFR1-3 that was evaluated in a phase I study (NCT01948297) in patients with iCCA (n = 9) with *FGFR2* translocation (n = 5), *FGFR1* translocation (n = 1), *FGFR2* mutation (n = 1), *FGFR2* activating deletion (n = 1), and *FGFR3* mutation (n = 1). PRs were achieved in 22% (1 *FGFR2* fusion, 1 *FGFR2* activating deletion) and 44% had SD (all *FGFR2* translocation) [56].

### 3.9 Ponatinib

A phase II (NCT02265341) is registered to determine the efficacy of ponatinib, a type IIA multi-target tyrosine kinase inhibitor, in patients with advanced cholangiocarcinomas harboring *FGFR2* fusions [57]. Ponatinib was further evaluated in patients (n = 12) with advanced biliary tract cancer (83.3% iCCA; 16.7% gallbladder) with *FGFR2* alterations (83.3% *FGFR2* translocations) who received prior chemotherapy. mPFS was 2.4 months (95% CI, 1.9–9.2) with a mOS of 15.7 months (95% CI, 6.1- not estimable) [58]. The multi-targeted activity of ponatinib leads to a broad profile of targets resulting in limited anti-tumor responses. Nevertheless, the broad profile of the agent might be beneficial in patients with resistance mutations to other FGFR inhibitors.

The outcomes of a study by Lin Q et al. support this and suggest a role for ponatinib in overcoming therapy resistance in cholangiocarcinoma. Ponatinib was developed to overcome resistance to Abl T315I and EGFR T790 M, based on gatekeeper mutations in kinase domains after the initial response to ABL and EGFR kinase inhibitors agents [59].

### 3.10 RLY-4008

RLY-4008 is a highly-selective potent next-generation FGFR2 inhibitor developed to target driver alterations and *FGFR* resistance mutations. A phase I/II study ReFocus (NCT04526106) was conducted to evaluate RLY-4008 in patients (n = 38) with cholangiocarcinoma harboring *FGFR2* fusions/rearrangements who did not receive prior FGFR inhibitors. A confirmed ORR of 57.9% (95% CI, 40.8–73.7) was achieved and a DCR of 94.7% was observed [60]. These outcomes suggest that expanding the repertoire of current FGFR inhibitors provides appropriate alternative options in patients with non-fusion alterations and secondary resistance.

### 3.11 AZD4547

AZD4547 is a type I1/2A FGFR1-3 inhibitor. Currently, a phase Ib study BISCAY (NCT02546661) is ongoing to evaluate, among other things, AZD4547 in patients with MIBC with confirmed *FGFR3* mutation or fusions progressed on prior therapy. Another phase Ib study (NCT05086666) recruits patients with urothelial cancer exhibiting *FGFR2/3* gene alterations to evaluate AZD4547.

### 3.12 Other FGFR inhibitors

A phase I study (NCT05242822) evaluates KIN-3248 in patients with advanced tumors, including iCCA and urothelial carcinoma, harboring *FGFR2* and/or *FGFR3* genomic alterations**.** Moreover, a phase II study (NCT04353375) is reported to evaluate HMPL-453 in patients with advanced iCCA harboring *FGFR2* fusions. Furthermore, ICP-192 is evaluated in a phase II study (NCT04492293) recruiting patients with bladder urothelial cancer harboring *FGFR2* genomic aberrations. Lastly, LY2874455, a typeI1/2B pan-FGFR blocker, and nintedanib (BIBF1120), a type I multi-kinase inhibitor, are being evaluated in studies (NCT01212107 and NCT02278978, respectively).

## 4 (Pre) clinical evidence of FGFR inhibitor resistance

Long-term therapeutic efficacy can be limited for FGFR inhibitors in treating cholangiocarcinomas and urothelial carcinomas harboring specific genomic aberrations. Multiple factors contribute to the challenge of treating these cancers in clinical studies. First, an initial lack of response to therapy might exist. Moreover, the progression of the disease after the initial treatment response is observed. Acquired resistance develops as a result of, for instance, secondary gatekeeper mutations within the FGFR kinase protein through heterogeneity among tumor subclones or (hyper)activation of downstream or alternative compensatory signaling pathways [17]. Gatekeeper mutations modulate the accessibility of the FGFR inhibitors to the ATP-binding domain on a protein kinase of, for instance, FGFR2 or FGFR3, and thus might prevent FGFR inhibitors' binding [3]. This occurs through steric hindrance in the ATP-binding pockets that the closed conformation of the kinase domains is incompatible with the binding of the inhibitor. This results in reduced sensitivity to FGFR-targeted therapy and clinically-acquired resistance [61].

As previously discussed, most of the FGFR inhibitors bind reversibly to the ATP-binding pockets of the receptors. Reversible ATP-competitive FGFR inhibitors mainly bind the hinge regions of the FGFR ATP-binding pockets. It inhibits all 4 isoforms as well (FGFR1-4). Mutations in these binding sites result in drug resistance and are thus common with reversible ATP-competitive kinase inhibitors [12]. Inhibitors that bind covalently to the kinase domains inhibit kinase activity irreversibly and may be superior to conventional inhibitors. For instance, futibatinib demonstrated a different binding mode to FGFR than AZD4547, infigratinib, erdafitinib, pemigatinib, or even irreversible PRN1371 [12]. Moreover, preclinical studies demonstrated that following treatment with futibatinib resulted in fewer resistant clones.

A clinical study is evaluating futibatinib and reported activity of the agent in patients diagnosed with iCCA harboring *FGFR2* fusions who developed resistance to BGJ398 or Debio1347, with some patients having secondary FGFR2 kinase mutations [62]. The agent thus demonstrated clinical benefits in iCCA by inhibitory effects against most secondary-acquired resistance mutations in FGFR kinase domains. These findings suggest a role of FGFR2-translocated cholangiocarcinoma after progression following ATP-competitive FGFR inhibitors; however, the agent failed to overcome gatekeeper mutations in cysteine residues [3]. Futibatinib interacts with the cysteine in the phosphate-binding loop (P-loop) of the ATP-binding pockets of the FGFR protein kinase domain. However, the advantage of the agent is that it can target different conformations of the P-loop. Therefore, they are less affected by mutations in the ATP-binding pockets, which generally reduce the binding affinity of reversible ATP-competitive FGFR inhibitors. In other words, the unique mechanism of action of futibatinib is promising in overcoming acquired resistance to ATP-competitive inhibitors.

Resistance to other irreversible kinase inhibitors may arise due to cysteine residue mutations mediating the covalent bonds [62]. Clinical resistance to the FDA-approved FGFR inhibitors erdafitinib and pemigatinib is not yet reported. However, preclinical data demonstrated reduced activity of the two drugs in *FGFR2* mutants related to drug resistance [12]. Moreover, patients (n = 3) with iCCA who were previously treated with NVP-BGJ398 acquired a point mutation in the FGFR2 protein kinase domain and showed resistance and progression of disease [63]. Bladder cancer cell lines with FGF3 dependency developed resistance to NVP-BGJ398 by switching signaling to alternative tyrosine kinase receptor(s) [64]. These outcomes suggest resistance mechanisms against infigratinib.

## 5 Future perspectives for treating urothelial carcinomas and cholangiocarcinomas

Patients diagnosed with FGFR-altered cholangiocarcinoma and urothelial carcinoma have a poor prognosis and novel therapeutic strategies are greatly warranted [65]. Current front-line therapy consisting of chemotherapy with gemcitabine and cisplatin and with durvalumab offers a median PFS of 7.2 months and OS of 12.8 months in cholangiocarcinoma [15]. Progression is often seen, and second-line treatment options or further are limited (to specific subtypes). An OS of 14–15 months is achieved in urothelial cancer after first-line cisplatin-based chemotherapy; however, the durability of the response remains poor [66]. Significant clinical efforts are made to discover targetable genetic aberrations in cancers, altering the treatment paradigm of the cholangiocarcinomas and urothelial cancers toward targeted therapy both in the first-and second-line setting.

A retrospective cohort evaluated pemigatinib, derazantinib, futibatinib, pazopanib, ponatinib, erdafitinib, RLY-4008, infigratinib, and AZD-4547 in patients (n = 88) with advanced or metastatic cholangiocarcinoma exhibiting *FGFR* alterations. mPFS on initial FGFR was 6.6 months (95% CI, 5.5–8.3), mPFS for chemotherapy or targeted treatment (n = 32) following FGFR treatment was 2.1 months (95% CI, 1.6–5.7), and mPFS for patients who received a second FGFR inhibitor (n = 16) was 3.7 months (95% CI, 1.5-not estimable). The latter achieved an OS of 8.6 months (95% CI, 5.5-not estimable) compared to 2 months (95% CI, 0.7–3.1 months) for patients not receiving any therapy and 8.7 months (95% CI, 6.0–20.9) for patients receiving chemotherapy as second treatment. However, DCR was higher in patients who received a second FGFR inhibitor (69% compared to 44% in patients who received other second therapy), suggesting that more cycles of FGFR inhibition provide clinical benefits in refractory patients [67].

It has become clear that targeting FGFR pathway signaling in urothelial cancer and cholangiocarcinoma has enhanced patient outcomes in defined *FGFR*-altered individuals. Clinical studies in biliary tract cancers demonstrate superior clinical responses to FGFR inhibitors in patients exhibiting *FGFR2* fusions and rearrangements compared to other *FGFR* aberrations where marginal success was observed. Moreover, iCCA has the most potential of having actionable genomic targets. Current FDA approvals and recommendations reflect this discordance as pemigatinib, infigratinib, and futibatinib are the only FDA-approved FGFR inhibitors for cholangiocarcinoma positive for *FGFR2* fusions or rearrangements. The same trend is observed for urothelial carcinoma, where clinical studies demonstrate the best activity in cancers harboring *FGFR2/3* mutations. For this group of cancer, erdafitinib is approved for first-line treatment. However, these subpopulations only represent a small group of patients diagnosed with urothelial carcinomas and cholangiocarcinomas. Therefore, novel drivers and targetable genetic alterations are urgently needed, while current strategies are further investigated in the clinical setting as front-line therapy or part of a combination treatment to overcome resistance mechanisms.

FGFR aberrations have been tied to FGFR inhibitor resistance and as a result, the cancers harboring these aberrations are challenging to treat, causing significant concern. Given the tumor molecular heterogeneity associated with drug sensitivity and acquired resistance, genomic profiling is becoming more important in informing and sequencing treatment strategies [28]. Proper selection of patients who will benefit from FGFR-targeted treatment and enrollment in clinical trials is needed. Next generation sequencing (NGS) of tumor tissue before and after progression of FGFR treatment and analysis of matched circulation tumor DNA (ctDNA) upon progressions. ctDNA analysis revealed novel mutations in the *FGFR2* kinase domain and resistance mechanisms in FGFR inhibitor treatment. Sequencing of FGFR inhibitors, guided by serial biopsies and ctDNA, may extend the effect of FGFR inhibition. In addition, revealing genotype-phenotype correlations for sensitivity to drugs might be crucial to inform personalized medicine. Moreover, gaining knowledge of the spectrum of activity of FGFR inhibitors against acquired *FGFR2* mutations may develop novel strategies to overcome resistance.

In addition, overcoming acquired resistance with combination strategies and/or selective dual-targeted FGFR inhibitors simultaneously inhibit several synergistic targets, thereby circumventing the activation of compensatory pathways related to drug resistance. For instance, preclinical evidence demonstrates pERBB3 as an adaptive resistance mechanism to FGFR inhibitor in bladder cancer with *FGFR3* fusion. This study demonstrated that combined treatment consisting of a pan-ERBB inhibitor could sensitize cancer cells to FGFR inhibitors and inhibit compensatory pathways after FGFR therapy [68].

Another challenge in the FGFR inhibitor treatment is the management of adverse events that reduce the optimal efficacy (Table 2). These drug-associated toxicities result in dose reductions, interruptions, and even discontinuation of the FGFR targeted therapy. Prevention and management of these side effects are crucial for improving the tolerance and adherence to these drugs.

**Table 2:** **FGFR inhibitor safety.** Overview of some of the most common adverse events of FGFR inhibitors.

FGFR inhibitor	Reference	Hyperphosphatemia All grades/Grades 3–4	Alopecia All grades/Grades 3-4	Diarrhea All grades/Grades 3-4	Fatigue All grades/Grades 3-4	Nausea All grades/Grades 3-4	Dysgeusia All grades/Grades 3-4
Pemigatinib	[21] n = 108	58.5%/0%	49.7%/0%	46.9%/3.4%	43.5%/5.4%	41.5%/2%	40.8%/0%
	[24] n = 31	77.4%/3.2%	54.8%/0%	41.9%/0%	35.5%/0%		32.3%/0%
	[25] n = 100	64%/0%	32%/0%	40%/0%	29%/6%		
Futibatinib	[27] n = 170	81.2%/22.4%	19.4%/0%	32.9%/0.6%	25.3%/5.3%	28.2%/0%	10.6%/0%
	[28] n = 103	85%/30%	33%/0%	28%/0%	25%/6%	12%/2%	18%/0%
Infigratinib	[34] n = 71	73.2%/12.7%	38%/0%		49.3%/0%		
	[35] n = 108	77%/10%	38%/0%	24%/3%	40%/4%	19%/1%	31%/0%
	[36] n = 67	46.3%/1.5%	31.3%/0%	19.4%/3%	37.3%/7.5%	28.4%/4.5%	20.9%/0%
	[37] n = 67	38.8%/0%	31.3%/0%		38.8%/7.5%	28.4%/4.5%	22.4%/0%
Erdafitinib	[40] n = 65	65%/0%	14%/0%	22%/0%	55%/0%	25%/0%	31%/0%
	[41] n = 187	64%/0%	18%/0%	20%/0%	13%/0%	16%/0%	26%/0%
	[42] n = 99	77%/2%	29%/0%	51%/4%	32%/2%	20%/1%	37%/1%
	[43] n = 96	69%/2%		42%/3%			
	[44] n = 101	76%/2%	34%/0%	50%/4%	31%/2%	21%/1%	39%/2%
Derazantinib	[48] n = 80	5%/0%		29%/0%	58%/6%	54%/1%	11%/0%
	[49] n = 29	75.9%/10.3%	24.1%/0%	20.7%/0%	34.5%/3.4%	44.8%/0%	31%/0%
	[50] n = 103	37%/4%			31%/5%	30%/0%	
Dovitinib	[52] n = 13			77%/0%	85%/15%	46%/0%	54%/0%
Rogaratinib	[54] n = 86	45.3%/0%	23.3%/0%	55.8%/4.7%	24.4%/2.3%	32.6%/2.3%	15.1%/0%
	[55] n = 126	60%/1%	20%/0%	49%/2%	24%/9%	28%/2%	20%/0%

## 6 Conclusion

In recent years, the urothelial and cholangiocarcinoma treatment landscape has rapidly evolved. Erdafitinib is already approved for first-line treatment in urothelial cancers exhibiting *FGFR2* or 3 mutations. In cholangiocarcinoma, FGFR inhibitors are promising in contributing to second-line therapy following conventional treatment and their role in the first-line setting is hopeful. Phase III trials FIGHT-302 [69] and FOENIX-CAA3 [70] aim to achieve an mPFS that outperforms the 7.2-month mPFS achieved with the current first-line treatment. These trials are promising since, in contrast to previous studies (pemigatinib mPFS 7.0 months and futibatinib 9.0 months), they recruit a more homogenous group of patients (exclusively genetically homogenous iCCA). Additional FDA approval of other FGFR inhibitors will likely be forthcoming due to the impressive results from numerous (pre)clinical studies. Future efforts will be made by appropriate use and sequencing of current therapies and research elucidating resistance mechanisms during targeted therapy, contributing to developing novel agents and combination therapies. Comprehensive genomic profiling may play a key role in molecular-based patient stratification, developing novel FGFR inhibitors, and combination therapy.

## Funding

This research did not receive any specific grant from funding agencies in public, commercial or not-for-profit sectors.

## Author contribution statement

All authors listed have significantly contributed to the development and the writing of this article.

## Data availability statement

No data was used for the research described in the article.

## Declaration of competing interest

The authors declare the following financial interests/personal relationships which may be considered as potential competing interests:Cinzia Solinas received travel grants from Eli Lilly outside the present work.All other authors have no conflict of interest to declare related to the present topic.
